# 530 Utilization of Advanced Practice Provider for Quality Virtual Burn Care

**DOI:** 10.1093/jbcr/irad045.127

**Published:** 2023-05-15

**Authors:** Brittany Ayers, Bryan Bader

**Affiliations:** Regions Hospital, St. Paul, Minnesota; Regions Hospital, St. Paul, Minnesota; Regions Hospital, St. Paul, Minnesota; Regions Hospital, St. Paul, Minnesota

## Abstract

**Introduction:**

From the plains of North Dakota to the lakes of Minnesota and beyond, for our patient population, telemedicine has grown to be an important burn care delivery method. Specialized burn care is essential to ensure successful patient outcomes from burn injury. Our institution utilizes an advanced practice provider (APP) either physician assistant or nurse practitioner to lead the telemedicine care instead of the burn surgeon. Given most of the burn care is non-operative management, an APP can deliver burn expertise and high-quality care to the burn patient.

**Methods:**

A retrospective chart review performed via data extraction from the electronic medical record at a single ABA-verified Burn Center. Dates of review ranged from January 1, 2022, through June 30, 2022. Patient population was Regions Hospital Outpatient Burn Clinic and Telemedicine Clinic volumes. Data review included patient location, mode of visit (in-person or virtual), and time to >95% healed. Inclusion was acute burns of all ages and mechanisms of injury. Exclusion was non-burn wounds, scar management and frostbite patients.

**Results:**

A total of 1300 (87%) of all outpatient visits performed by APPs with 328 (25%) of these visits conducted via telemedicine. Varying by month, 7-13% of patients were treated exclusively via telemedicine not requiring any in-person burn care. When comparing the patient distance from the burn center (1-50 miles and >50 miles) and time to >95% healed, there was no correlation. Patients who were treated exclusively via telemedicine, lived an average of 205 (5-580) miles from the burn center, had an average TBSA of 2.2 (0.25-55) % and had an average of 18.3 (7-62) days until 95% healed. Compared to patients treated in-person only or combination of in-person and telemedicine, lived an average of 91 (1-631) miles, had an average TBSA of 2.5 (0.25-67) % and had an average of 24.6 (5-80) days until 95% healed.

**Conclusions:**

A burn telemedicine program led by APPs can facilitate remote expert burn care, all while maintaining standard of care. Limitations were a narrow review window, single center retrospective analysis, and the exact date for time to 95% healed is difficult to determine due to interval follow up windows. Our goal is to further review and analyze our telemedicine and clinic data to optimize burn care delivery with the utilization of APPs, investigate quality of care indicators within telemedicine and reduce transfers and travel for specialized burn care.

**Applicability of Research to Practice:**

APP led telemedicine improves surgeon availability to do hands on treatment in the Burn Center and allows for outreach and specialized expertise to be delivered across remote distances. It allows for the ability to triage patients and optimize burn clinic resources. Telemedicine can be performed in any location and reduce travel. Telemedicine is reimbursable. Barriers include wound care supply access, technological limitations, state licensing and institutional credentialing.

